# U-shaped association between fasting blood glucose and suicide attempts in Chinese patients with first-episode drug-naïve major depressive disorder

**DOI:** 10.1186/s12888-024-05818-9

**Published:** 2024-05-21

**Authors:** Junjun Liu, Guangya Zhang, Fengnan Jia, Hsinsung Yuan, Qingyuan Wang, Chuanwei Li, Ruchang Yang, Yan Yue, Xiaobin Zhang, Gang Ye, Zhe Li, Xiangdong Du, Xiangyang Zhang

**Affiliations:** 1 Nanjing Meishan Hospital, Nanjing, 210041 PR China; 2 Department of Psychiatry, Suzhou Guangji Hospital, The Affiliated Guangji Hospital of Soochow University, Suzhou, 215137 PR China; 3 Medical College of Soochow University, Suzhou, 215137 PR China; 4 Clinical Medical Department, the Second Clinical Medical College, Nanjing Medical University, Nanjing, 211166 PR China; 5 CAS Key Laboratory of Mental Health, Institute of Psychology, Chinese Academy of Sciences, 16 Lincui Road, Chaoyang District, Beijing, 100101 PR China

**Keywords:** Association, Fasting blood glucose, Suicide attempt, Major depressive disorder, Non-linear relationship

## Abstract

**Background:**

Evidence regarding the relationship between fasting blood glucose (FBG) and suicide attempts (SA) in patients with major depressive disorder (MDD) was limited. Therefore, the objective of this research was to investigate whether FBG was independently related to SA in Chinese patients with first-episode drug-naïve (FEDN) MDD after adjusting for other covariates.

**Methods:**

The present study was a cross-sectional study. A total of 1718 participants (average age: 34.9 ± 12.4 years, 65.8% females) with FEDN MDD were involved in a hospital in China from September 2016 to December 2018. Multiple logistic regression analysis and smooth curve fitting were used to estimate the association between FBG and the risk of SA. The threshold effect was examined by the two-piecewise linear regression model. Interaction and stratified analyses were conducted according to sex, education, marital status, comorbid anxiety, and psychotic symptoms.

**Results:**

The prevalence of SA in patients with FEDN MDD was 20.1%. The result of fully adjusted binary logistic regression showed FBG was positively associated with the risk of SA (odds ratio (OR) = 1.62, 95% CI: 1.13–2.32). Smoothing plots also revealed a nonlinear relationship between FBG and SA, with the inflection point of FBG being 5.34 mmol/l. The effect sizes and the confidence intervals on the left and right sides of the inflection point were 0.53 (0.32–0.88, *P* = 0.014) and 1.48 (1.04–2.10, *P* = 0.030), respectively.

**Conclusions:**

A U-shaped relationship between FBG and SA in FEDN MDD patients was found, with the lowest risk of SA at a FBG of 5.34 mmol/l, indicating that both the lower and higher FBG levels may lead to an increased risk of SA.

## Introduction

Major depressive disorder (MDD) is a highly prevalent psychiatric disorder characterized by a sustained period of at least two weeks of low mood, diminished interest or pleasure in activities, persistent fatigue or loss of energy, significant changes in appetite or weight, psychomotor agitation or retardation, insomnia or hypersomnia, and recurrent thoughts of death or suicide [1]. The presence of suicide-related behaviors in individuals with MDD is a major concern due to its alarming consequences. Even with adequate treatment for depression, previous epidemiological studies have reported that approximately 15% of individuals with MDD eventually die by suicide [2]. A comprehensive study conducted across four counties and a large urban region in the United Kingdom revealed that 77% of individuals who died by suicide had a mental condition at the time of death, with MDD being the most common condition observed in 63% of cases [3]. Suicide attempts (SA) have consistently been identified as a significant predictor of completed suicides. Previous research has shown that approximately 16–33.7% of individuals with MDD have made at least one suicide attempt in their lifetime [4, 5], which is approximately 20 times higher than the general population [6]. In China, the prevalence of suicide attempts among individuals with MDD ranges from 7.3 to 48.4% [7, 8]. Despite these alarming statistics, the exact causes of suicide attempts in individuals with MDD remain unclear. Therefore, it is crucial to identify and understand the relevant risk factors associated with suicide attempts in order to effectively screen for and prevent suicide in this vulnerable population.

The causes of suicidal behavior in patients with MDD are multifactorial and can be attributed to various biological, psychosocial, and environmental factors. Several variables have been identified to significantly impact the epidemiologic characteristics of suicidal behavior in MDD patients. For instance, being male, younger in age at onset, and having severe depressive pathology are all associated with a higher risk of suicidal behavior in MDD patients [9–11]. Additionally, physical illness, drug or alcohol abuse or dependence, a prior history of suicidal attempts, and physical inactivity further contribute to this risk. Furthermore, previous research has explored the potential role of fasting blood glucose (FBG) in the neurobiological mechanism underlying suicidal behavior [12, 13]. While the relationship between FBG and suicidal behavior in adults is still debatable, some studies have yielded interesting findings. For example, a study conducted in Korea found that diabetic patients had higher rates of depression, suicidal ideation, and suicide attempts compared to individuals without diabetes [14]. Another study discovered that young MDD patients with suicidal behavior had significantly higher levels of FBG compared to those without such behavior [15]. Moreover, researchers reported that MDD patients with suicidal behavior exhibited higher FBG levels and lower glucose metabolism rates in specific brain regions [16]. Despite these findings, there is inconsistent evidence regarding whether glucose disturbances contribute to the risk of suicidal behavior in MDD patients. Some studies have failed to establish a statistically significant association between blood glucose levels and suicidal behavior in MDD patients [17, 18]. Additionally, a recent study indicated that, while FBG may serve as a potential biomarker for suicidal behavior in men, the correlation between FBG and suicidal behavior in women with MDD is not significant [19]. It is important to acknowledge the challenges in comprehending or comparing these results due to the discrepancies between studies. Factors such as racial differences, variations in study populations (including general population samples and patients), differences in hospitalization histories, the influence of antidepressant medication, and variations in statistical methods utilized may contribute to these inconsistencies.

First-episode drug-naive (FEND) MDD patients, who have not yet been treated with medications for their depression, provide a unique opportunity for researchers to investigate the relationship between SA and FBG in MDD patients without the confounding effects of lifestyle changes and medication treatments. By excluding these factors, the study aims to avoid interference from other variables such as the duration of the illness and the presence of other medical conditions that could potentially affect the results [20]. Although previous research in this area has yielded conflicting and complex findings, there is evidence to suggest a close connection between FBG and SA in MDD patients. As Asians and Westerners have distinct physical characteristics due to their different racial backgrounds, it is important to explore this association among FEND MDD patients in the Chinese population, as there is currently limited knowledge in this area. Therefore, the objective of this study is to assess the correlation between FBG and SA in a relatively large sample of FEND MDD patients in China.

## Materials and methods

### Subjects

The present cross-sectional study was conducted from September 2016 to December 2018 in the First Hospital of Shanxi Medical University, a general hospital in Taiyuan, Shanxi Province, China. A total of 1718 FEND patients with MDD (588 males and 1130 females; age range of 18–60 years) were recruited consecutively.

Recruitment criteria included: (1) being Han Chinese and between the ages of 18 and 60; (2) never having received antidepressant treatment in the past; (3) meeting the Diagnostic and Statistical Manual of Mental Disorders (DSM) IV-TR criteria for MDD; (4) current depressive symptoms being the first episode; and (5) with the 17-item HAMD score > = 24. Exclusion criteria include the following: (1) with severe physical diseases such as cancer, ongoing infections, epilepsy, brain injury, diabetes, and stroke (*n* = 9); (2) pregnancy or lactating women (*n* = 10); (3) alcohol or drug abuse except for tobacco smoking (*n* = 9); (4) meeting any other major Axis I disorders such as schizophrenia, schizophrenic affective disorder, bipolar disorder and other psychotic disorders (*n* = 15); (5) refusal of taking part in this study (*n* = 21), (6) interviewing difficult or unreliable (*n* = 5), (7) and other undocumented reasons (*n* = 9). The study was approved by the Institutional Review Board (IRB) of Shanxi Medical University (No. 2016-Y27) and performed in accordance with the Declaration of Helsinki. All subjects in this study signed a written informed consent form to participate in this study.

### Socio-demographic characteristics and anthropometric measurements

The patients’ socio-demographic parameters and general data, such as age, sex, education level, marital status, age at onset, and duration of illness, were gathered using a structured, self-designed questionnaire. Weight and height measurements, as well as systolic blood pressures (SBP) and diastolic blood pressures (DBP), were acquired using standardized procedures and calibrated equipment. The weight in kilograms (kg) divided by the square of the height in meters (kg/m^2^) yields a body mass index (BMI).

A face-to-face interview was used to collect information about individuals’ suicide attempt histories. All participants were asked the question, “Have you ever attempted suicide in your entire lifetime?” which was derived from the WHO/EURO multiple-center study [21]. Those participants who answered “yes” to the question were considered to have had suicidal attempts. Subsequent inquiries focused on detailing the frequency, methods, and precise timing of suicide attempts. Where participant responses were ambiguous or unclear, further information was garnered through interviews with family members, relatives, or friends. A total of 346 patients diagnosed with MDD were reported to have attempted suicide during their initial depressive episode, with 111 of these incidents occurring within the past month. The breakdown of these attempts included one patient with four attempts, two patients with three attempts, 26 with two attempts, and 317 with one attempt. The individuals were split into two groups based on whether they had attempted suicide or not: the suicide attempts group (SA = 346) and the non-suicide attempts group (NSA = 1372).

### Clinical interview assessments

Each patient with MDD acquired a consensus diagnosis from at least two experienced psychiatrists based on the Chinese version of the Modified Structured Clinical Interview for DSM-IV-TR Criteria (SCID-I/P).

The 17-item Hamilton Rating Scale (HAMD-17) was used to evaluate the severity of depression. Eight of the 17 items on the scale are graded on a 5-point scale, from 0 (not present) to 4 (severe), while the other nine items are graded from 0 (none) to 2 (symptom-specific severity descriptor). A higher score on the scale, which has a total range of 0 to 52, suggests more severe depressive symptoms. The scale, which is extensively used in China, has been shown to have strong reliability and validity in previous studies [22].

Additionally, the Hamilton Anxiety Rating Scale (HAMA) was used to assess the severity of the current anxiety symptoms. The scale consists of 14 symptom-defined items, each of which is scored on a 5-point Likert scale from 0 (no symptoms) to 4 (severe symptoms), for a total score that can range from 0 to 56. The HAMA scale measures both psychic anxiety (e.g., mental agitation and psychological distress) and somatic anxiety (e.g., physical complaints related to anxiety). In this study, a cut-off point of 18 was employed to categorize participants into groups with or without anxiety symptoms [23].

The Positive and Negative Syndrome Scale (PANSS) was used to assess psychotic symptoms using its positive subscale. Each item was scored from 1 (absent) to 7 (extreme severity). As a result, the PANSS positive subscale’s total score ranged from 7 to 49. In this study, patients with a total positive PANSS subscale score of 15 or above were defined as having psychotic symptoms [24].

Before the study, two certified psychiatrists with at least five years of clinical experience received a training session on the use of these rating scales. Inter-observer reliability on the HAMA, HAMD, and PANSS positive subscales of total scores at repeated assessments throughout the study was maintained after training with a correlation coefficient of more than 0.8. In addition, they were blinded with regard to the patients’ clinical status.

### Blood samples

Between the hours of 6:00 and 9:00 a.m., serum samples from all patients were collected following an overnight fast. Subsequently, the hospital’s laboratory center received the entirety of the acquired blood samples. Prior to 11:00 a.m. on the same day, assessments were conducted to measure the concentrations of thyroid-stimulating hormone (TSH), free triiodothyronine (FT3), free thyroxine (FT4), thyroid peroxidase antibody (TPOAb), anti-thyroglobulin (TgAb), low-density lipoprotein cholesterol (LDL-C), high-density lipoprotein cholesterol (HDL-C), cholesterol (TC), triglycerides (TG), and fasting blood glucose (FBG).

### Statistics

A Kolmogorov-Smirnov one-sample test was used to determine the distribution of continuous variables. Normally distributed and non-normally distributed continuous variables were respectively reported by their means with standard deviations (SD) and median values with interquartile ranges (IQR), while categorical variables were presented as frequencies with proportions (%). We used a one-way ANOVA test (normal distribution), Kruskal–Wallis H test (skewed distribution), or χ2 (categorical variables) to test for differences among different FBG groups (Tertial). The association between FBG and SA was assessed with the use of multivariable logistic regression models. To quantify the strength of the association, unadjusted and adjusted odds ratios (ORs) with 95% confidence intervals (CIs) were estimated and reported. Multicollinearity between independent variables was calculated by the variance inflation factor (VIF), and those with a VIF greater than 5.0 were removed from the final model. The model included each covariate one at a time, and covariates were included as potential confounders in the final models if they changed the estimates of FBG on suicide attempt by more than 10% or were markedly associated with suicide attempt (*P* < 0.10) in the MDD patients [25]. Age, sex, education, duration of illness, HAMD, HAMA, TSH, A-TG, A-TPO, TC, HDL-c, TG, LDL-c, SBP, DBP, and psychotic symptoms were included in multivariate adjusted logistic regression models as confounders. To ensure the robustness of the data analysis, we performed a sensitivity analysis. We converted the FBG into a categorical variable and calculated the P value for trend. The relationship between FBG and SA was also explored using smoothing plots. A two-piecewise linear regression model based on the Generalized Estimating Equation (GEE) was applied to investigate the threshold effect according to the smoothing plot. To compare the different regression models, the log likelihood ratio (LLR) test was used. Then stratified analyses were performed by gender, education, marital status, comorbid anxiety, and psychotic symptoms, and their interactions were tested. A Bonferroni correction was also applied to control for inflation of Type 1 error rates with multiple tests. All of the analyses were conducted with the statistical software packages R (http://www.R-project.org, The R Foundation) and EmpowerStats (http://www.empowerstats.com, X&Y Solution, Inc, Boston, Massachusetts, USA). GraphPad Prism 8.0 was used to draw graphics. Two-tailed P values of 0.05 were considered to indicate statistical significance.

## Results

### Baseline characteristics of study participants

**Fig. 1:**
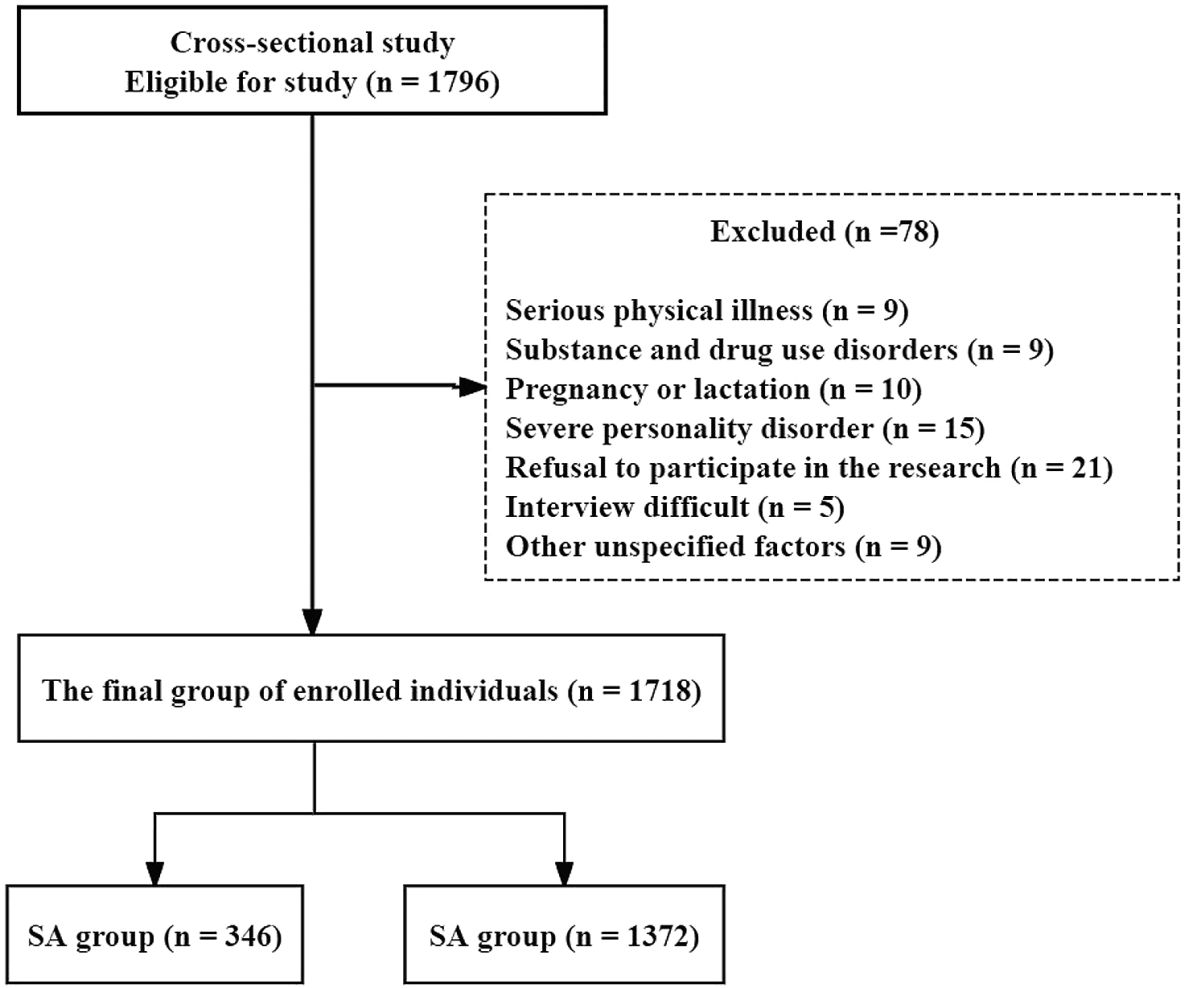
Flow chart of this study

**Fig. 2:**
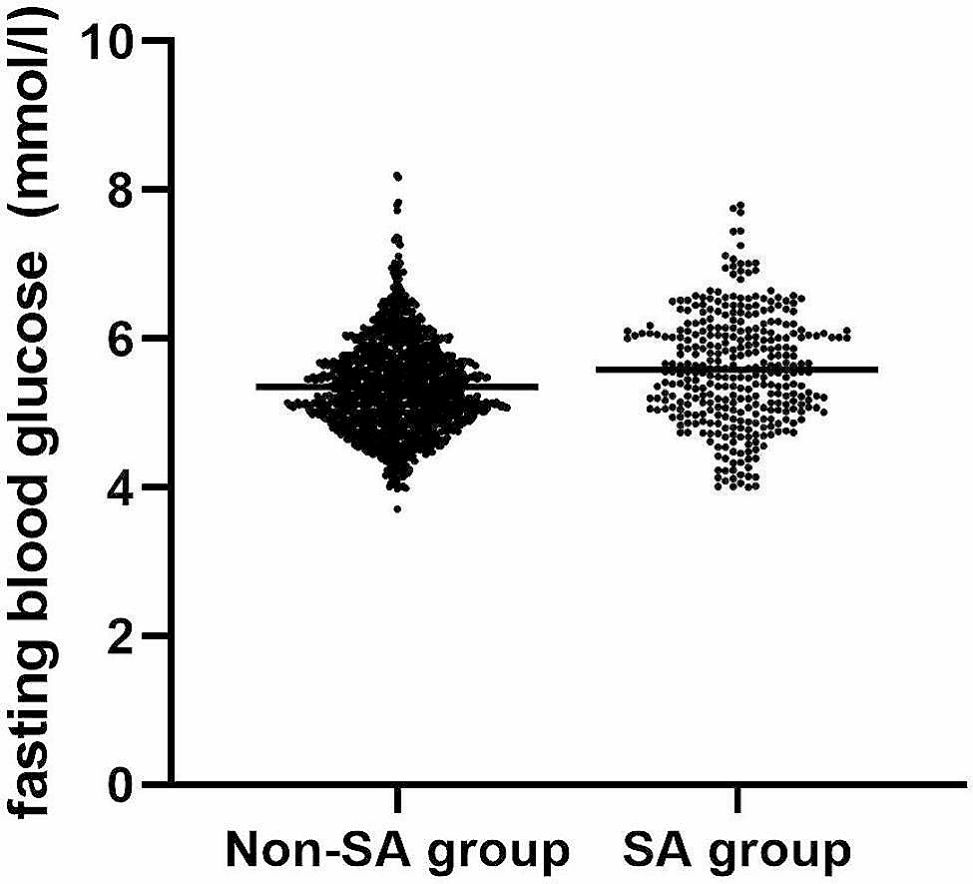
Distribution of fasting blood glucose levels in MDD patients with and without suicide attempts

A total of 1718 FEDN MDD patients were selected for the final data analysis after screening by inclusion and exclusion criteria (see Fig. 1 for a flow chart). In general, the average age of the participants was 34.9 ± 12.4 years, including 588 males (34.2%) and 1130 females (65.8%). In our study, a total of 346 patients had SA, and the prevalence of SA was 20.1%. The mean FBG level in FEDN MDD patients with SA was significantly higher than that in those without SA (5.49 ± 0.68 mmol/l vs. 5.37 ± 0.64 mmol/l; t=-6.15; *P* < 0.001; Fig. 2). We showed the baseline characteristics of these selected participants in Table 1 according to the tertiles of FBG. The subjects were divided into three equal parts according to the distribution of FBG. The FBG of the low group, middle group, and high group was 3.7-5.0 mmol/l (*n* = 571), 5.1–5.5 mmol/l (*n* = 572), and 5.6–8.2 mmol/l (*n* = 575), respectively. In the low, middle, and high FBG groups, the prevalence rates of SA were 16.5%, 15.4%, and 28.5%, respectively. Except for age, gender, age at onset, education, marital status, FT3 and FT4, other variables were significantly different among the different FBG tertiles (all P values > 0.05). Compared with the lowest tertile (T1), patients in the highest tertile (T3) tended to have a higher duration of illness, HAMD, HAMA, TSH, TGAb, TPOAb, TC, TG, LDL-c, BMI, systolic pressure, and diastolic pressure, but lower HDL-c. Moreover, the proportion of people who had SA, psychotic symptoms, and comorbid anxiety was higher in the highest group (T3). A p value < 0.05 (*p* < 0.017 in three comparisons after Bonferroni correction) was considered to indicate a statistically significant difference.

**Table 1:** Baseline characteristics of participants

Variables	FBG tertile (mmol/l)			*P*-value
	T1 (3.7-5.0)	T2 (5.1–5.5)	T3 (5.6–8.2)	
N	571	572	575	
Age (years)	34.0 ± 12.2	34.9 ± 12.4	35.7 ± 12.7	0.077 ^a^
Duration of illness (months)	4.0 (3.0–7.0)	6.0 (3.0–8.0)	6.0 (3.0–9.0)	< 0.001 ^c^
Age at onset (years)	33.9 ± 12.1	34.7 ± 12.2	35.5 ± 12.5	0.090 ^a^
HAMD	29.5 ± 2.9	30.2 ± 2.9	31.2 ± 2.8	< 0.001 ^a^
HAMA	20.4 ± 3.2	20.5 ± 3.4	21.4 ± 3.7	< 0.001 ^a^
TSH (uIU/ml)	3.8 ± 2.4	5.0 ± 2.0	6.4 ± 2.6	< 0.001 ^a^
TGAb (IU/l)	20.4 (14.3–30.1)	21.6 (13.9–37.0)	22.4 (15.4–89.8)	< 0.001 ^c^
TPOAb (IU/l)	16.7 (12.2–28.9)	16.6 (12.2–33.3)	19.5 (12.7–49.0)	< 0.001 ^c^
FT3 (pmol/l)	4.9 ± 0.7	4.9 ± 0.7	4.9 ± 0.7	0.866 ^a^
FT4 (pmol/l)	16.7 ± 3.2	16.6 ± 3.0	16.7 ± 3.1	0.850 ^a^
TC (mmol/l)	4.9 ± 1.0	5.3 ± 1.0	5.6 ± 1.1	< 0.001 ^a^
HDL-c (mmol/l)	1.3 ± 0.3	1.2 ± 0.3	1.1 ± 0.3	< 0.001 ^a^
TG (mmol/l)	2.1 ± 1.0	2.1 ± 0.9	2.3 ± 1.0	< 0.001 ^a^
LDL-c (mmol/l)	2.7 ± 0.8	3.0 ± 0.8	3.2 ± 0.9	< 0.001 ^a^
BMI (kg/m^2^)	24.1 ± 1.9	24.5 ± 1.9	24.5 ± 2.0	< 0.001 ^a^
Systolic pressure (mmHg)	116.1 ± 11.0	119.6 ± 10.5	122.7 ± 10.2	< 0.001 ^a^
Diastolic pressure (mmHg)	74.8 ± 6.7	75.6 ± 6.4	77.4 ± 6.8	< 0.001 ^a^
**Gender (n,%)**				0.332 ^b^
Male	209 (36.6%)	187 (32.7%)	192 (33.4%)	
Female	362 (63.4%)	385 (67.3%)	383 (66.6%)	
**Education (n,%)**				0.840 ^b^
Junior high school	127 (22.2%)	138 (24.1%)	148 (25.7%)	
Senior high school	260 (45.5%)	247 (43.2%)	253 (44.0%)	
College	153 (26.8%)	155 (27.1%)	141 (24.5%)	
Postgraduate	31 (5.4%)	32 (5.6%)	33 (5.7%)	
**Marital status (n,%)**				0.457 ^b^
Single	177 (31.0%)	166 (29.0%)	159 (27.7%)	
Marriage	394 (69.0%)	406 (71.0%)	416 (72.3%)	
Suicide attempts (n,%)				< 0.001 ^b^
No	477 (83.5%)	484 (84.6%)	411 (71.5%)	
Yes	94 (16.5%)	88 (15.4%)	164 (28.5%)	
Psychotic symptoms (n,%)				< 0.001 ^b^
No	532 (93.2%)	524 (91.6%)	491 (85.4%)	
Yes	39 (6.8%)	48 (8.4%)	84 (14.6%)	
**Comorbid anxiety (n,%)**				< 0.001 ^b^
No	131 (22.9%)	125 (21.9%)	82 (14.3%)	
Yes	440 (77.1%)	447 (78.1%)	493 (85.7%)	

*Note* The variables are presented as n (%) or the mean ± SD or median (quartile 1-quartile 3), FBG: fasting blood glucose; HAMD: 17-item Hamilton Rating Scale for Depression; HAMA: 14-item Hamilton Anxiety Rating Scale; TSH: thyroid-stimulating hormone; TGAb: thyroglobulin antibody; TPOAb: thyroid peroxidase antibody; FT3: free triiodothyronine; FT4: free thyroxine; TC: total cholesterol; HDL-c, high-density lipoprotein cholesterol; TG: triglyceride; LDL-c: low-density lipoprotein cholesterol; BMI: body mass index

^a^ one-way ANOVA test, ^b^ χ^2^ test, and ^c^ Kruskal–Wallis H test

### Univariate analysis

We listed the results of univariate analyses in Table 2. By univariate binary logistic regression, we found that gender, marital status, FT3, FT4, BMI, and postgraduate (1.04, 0.62–1.74 vs. ref) were not associated with SA (all P values > 0.05). We also found that age, duration of illness, age at onset, HAMD, HAMA, TSH, TGAb, TPOAb, TC, TG, LDL-c, systolic pressure, diastolic pressure, FBG, comorbid anxiety, and psychotic symptoms were positively associated with SA (all P values < 0.05). In contrast, univariate analysis showed that HDL-C, senior high school (0.71, 0.53–0.95 vs. ref), and college (0.69, 0.50–0.96 vs. ref) were negatively correlated with SA (all P values < 0.05). A p value < 0.05 (*p* < 0.017 in three comparisons after Bonferroni correction, with the junior high school group as the reference group, compared once with the senior high school group, college group, and postgraduate group, respectively) was considered to indicate a statistically significant difference.

**Table 2:** The result of univariate analysis for suicide attempts

Covariate	Statistics	OR (95%CI)	*P* value
Age (years)	34.9 ± 12.4	1.01(1.00, 1.02)	0.036
Duration of illness (months)	6.4 ± 4.7	1.03(1.01, 1.06)	0.007
Age at onset (years)	34.7 ± 12.3	1.01(1.00, 1.02)	0.038
HAMD	30.3 ± 2.9	1.36 (1.30, 1.43)	< 0.001
HAMA	20.8 ± 3.5	1.36 (1.31, 1.42)	< 0.001
TSH (uIU/ml)	5.1 ± 2.6	1.38 (1.31, 1.45)	< 0.001
TGAb (IU/l)	90.0 ± 238.3	1.01 (1.01, 1.02)	< 0.001
TPOAb (IU/l)	72.3 ± 163.8	1.03 (1.02, 1.04)	< 0.001
FT3 (pmol/l)	4.9 ± 0.7	1.02 (0.87, 1.20)	0.808
FT4 (pmol/l)	16.7 ± 3.1	1.00 (0.96, 1.04)	0.858
TC (mmol/l)	5.2 ± 1.1	1.73 (1.55, 1.94)	< 0.001
HDL-c (mmol/l)	1.2 ± 0.3	0.28 (0.18, 0.42)	< 0.001
TG (mmol/l)	2.2 ± 1.0	1.17 (1.04, 1.31)	0.008
LDL-c (mmol/l)	3.0 ± 0.9	1.45 (1.27, 1.66)	< 0.001
BMI (kg/m2)	24.4 ± 1.9	0.98 (0.93, 1.05)	0.622
Systolic pressure (mmHg)	119.5 ± 10.9	1.06 (1.04, 1.07)	< 0.001
Diastolic pressure (mmHg)	75.9 ± 6.7	1.07 (1.06, 1.09)	< 0.001
FBG (mmol/l)	5.4 ± 0.6	1.73 (1.45, 2.07)	< 0.001
**Gender**			
Male	588 (34.2%)	1.00 (Ref)	
Female	1130 (65.8%)	1.11 (0.86, 1.43)	0.416
**Education**			
Junior high school	413 (24.0%)	1.00 (Ref)	
Senior high school	760 (44.2%)	0.71 (0.53, 0.95)	0.022
College	449 (26.1%)	0.69 (0.50, 0.96)	0.027
Postgraduate	96 (5.6%)	1.04 (0.62, 1.74)	0.871
**Marital status**			
Single	502 (29.2%)	1.00 (Ref)	
Marriage	1216 (70.8%)	1.11 (0.86, 1.45)	0.420
**Comorbid anxiety**			
No	338 (19.7%)	1.00 (Ref)	
Yes	1380 (80.3%)	9.53 (5.16, 17.60)	< 0.001
**Psychotic symptoms**			
No	1547 (90.1%)	1.00 (Ref)	
Yes	171 (9.9%)	5.30 (3.81, 7.36)	< 0.001

### Results of unadjusted and adjusted binary logistic regression

In this study, we constructed three models to analyze the independent effects of FBG on SA (univariate and multivariate binary logistic regression). The effect sizes (odds ratio, OR) and 95% confidence intervals are listed in Table 3. In the unadjusted model, the model-based effect size can be explained as the difference in 1 mmol/l of FBG associated with the risk of SA. For example, the effect size of 1.73 for SA in the unadjusted model means that a difference in 1 mmol/l of FBG is associated with an increased 73% difference in the risk of SA (OR: 1.73, 95% CI, 1.45–2.07). In the minimum adjusted model (model I), the FBG was increased by 1 mmol/l and the risk of SA increased by 66% (OR: 1.66, 95% CI, 1.16–2.37). In the fully adjusted model (model II) for each additional 1 mmol/l of FBG, the risk of SA increased by 62% (OR: 1.62, 95% CI, 1.13–2.32). For the purpose of sensitivity analysis, we converted the FBG from a continuous variable to a categorical variable (Tertial of FBG), and the P-value for the trend of the FBG with categorical variables was consistent with the result of the FBG as a continuous variable in the different models (all P for trend < 0.05). In the fully adjusted model, the relative risks (95% CI) for participants in T1 (3.7-5.0 mmol/l) and T3 (5.6–8.2 mmol/l) were 1.63 (95% CI, 1.11–2.41, *P* = 0.014) and 1.43 (95% CI, 0.94–2.16, *P* = 0.091) respectively, when compared with those in T2 (5.1–5.5 mmol/l). Considering Type 1 error rates with multiple tests, according to the Bonferroni corrected standard, with the T2 group as the reference group, compared once with the T1 group and T3 group, respectively, then the significance level for each comparison in Table 3 should be adjusted to 0.05/2 = 0.025. The comparison p-value between the T1 group and the T2 group was 0.014, which was significant before correction and still significant after Boneroni correction (*p* < 0.025). However, the comparison p-value between the T3 group and the T2 group was 0.091, which was not significant before and after correction.

**Table 3:** Relationship between fasting blood glucose and suicide attempts in different models

Variable	Unadjusted model			Model I			Model II	
	OR (95%CI)	*P*-value		OR (95%CI)	*P*-value		OR (95%CI)	*P*-value
FBG	1.73 (1.45, 2.07)	< 0.001		1.66 (1.16, 2.37)	0.006		1.62 (1.13, 2.32)	0.009
FBG (tertile)								
T1	1.47 (1.00, 2.15)	0.049		1.49 (1.02, 2.18)	0.042		1.63(1.11, 2.41)	0.014
T2	Refrence			Refrence			Refrence	
T3	1.47 (0.98, 2.22)	0.064		1.46 (0.97, 2.20)	0.071		1.43 (0.94, 2.16)	0.091
*P* for trend	< 0.001			< 0.001			0.010	

*Abbreviations* CI, confidence interval; T1 (3.7-5.0 mmol/l); T2 (5.1–5.5 mmol/l); T3 (5.6–8.2 mmol/l); Unadjusted Model adjusted for none; Model I adjusted for age, sex; Model II adjusted for age, sex, education, duration of illness, HAMD, HAMA, TSH, A-TG, A-TPO, TC, HDL-c, TG, LDL-c, SBP, DBP

### Results of nonlinearity of FBG and SA

**Fig. 3:**
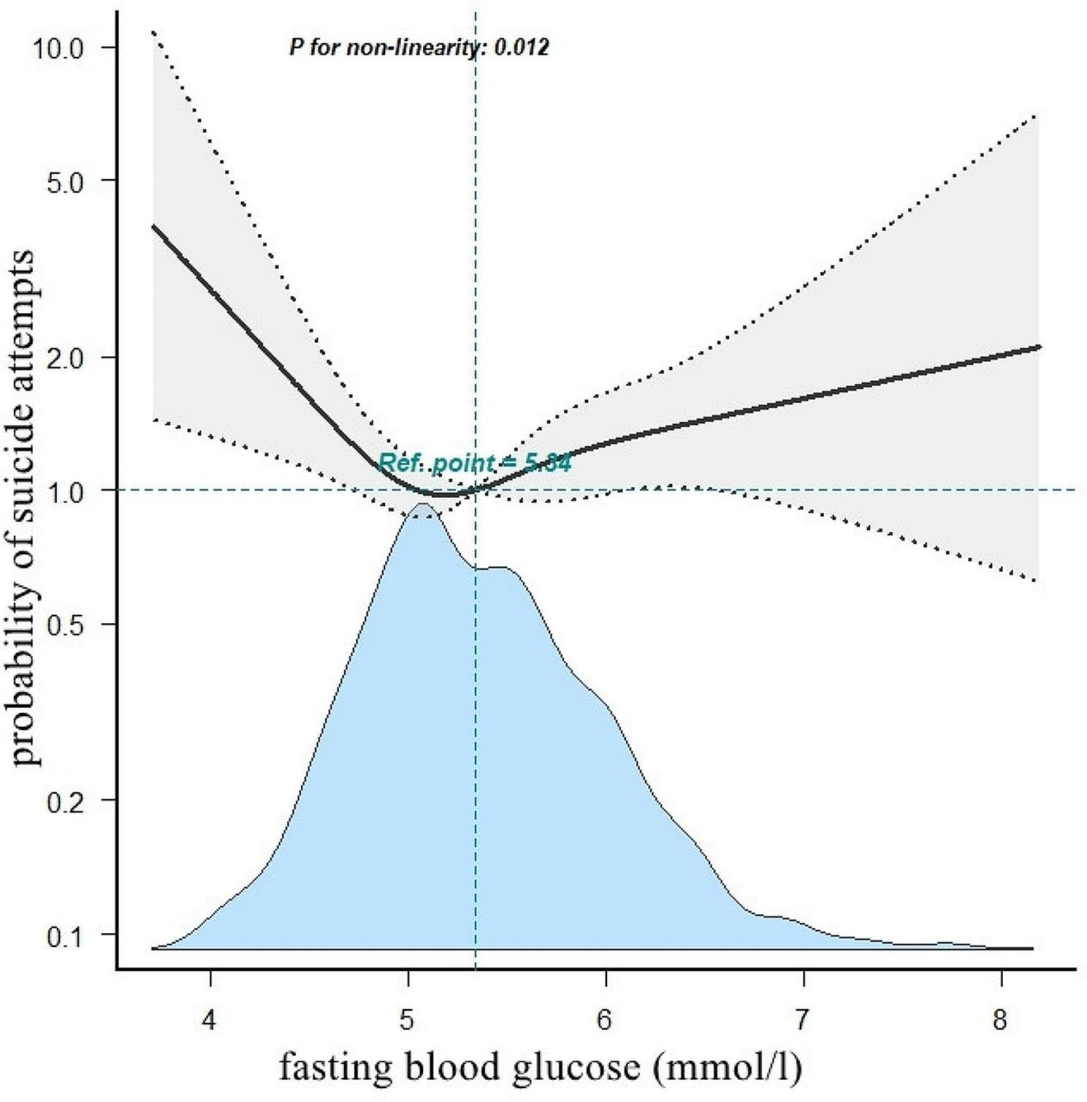
The relationship between fasting blood glucose and the probability of suicide attempts. A nonlinear relationship between fasting blood glucose and the probability of suicide attempts was observed after adjusting for age, sex, education, duration of illness, HAMD, HAMA, TSH, A-TG, A-TPO, TC, HDL-c, TG, LDL-c, SBP, DBP, and psychotic symptoms (P for non-linearity < 0.05)

In the present study, we analyzed the nonlinear relationship between FBG and SA (Fig. 3). The smooth curve fitting presented a U-shaped nonlinear relationship between FBG and SA after adjusting for age, sex, education, duration of illness, HAMD, HAMA, TSH, A-TG, A-TPO, TC, HDL-c, TG, LDL-c, SBP, DBP, and psychotic symptoms (P for non-linearity < 0.05). We used both binary logistic regression and two-piecewise binary logistic regression to fit the association and select the best fit model based on the log likelihood ratio test. Because the P for the log likelihood ratio test was less than 0.05, we chose two-piecewise binary logistic regression for fitting the association between FBG and SA because it can accurately represent the relationship. By using a two-piecewise binary logistic regression and a recursive algorithm, we calculated the inflection point to be 5.34 mmol/l. On the left side of the inflection point, the effect size and 95% CI were 0.53 and 0.32–0.88 (*P* = 0.014), respectively. On the right side of the inflection point, the effect size and 95% CI were 1.48 and 1.04–2.10 (*P* = 0.030), respectively (Table 4).

**Table 4:** The results of two-piecewise Logistic regression model

Inflection point of FBG	Effect size (OR)	95%CI	*P*
< 5.34 (mmol/l)	0.53	0.32 to 0.88	0.014
>= 5.34 (mmol/l)	1.48	1.04 to 2.10	0.030
Log likehood ratio test			0.006

Effect: Suicide attempts; Cause: FBG (mmol/l); adjusted for age, sex, education, duration of illness, HAMD, HAMA, TSH, A-TG, A-TPO, TC, HDL-c, TG, LDL-c, SBP, DBP

### Subgroup analyses of FBG and SA

**Fig. 4:**
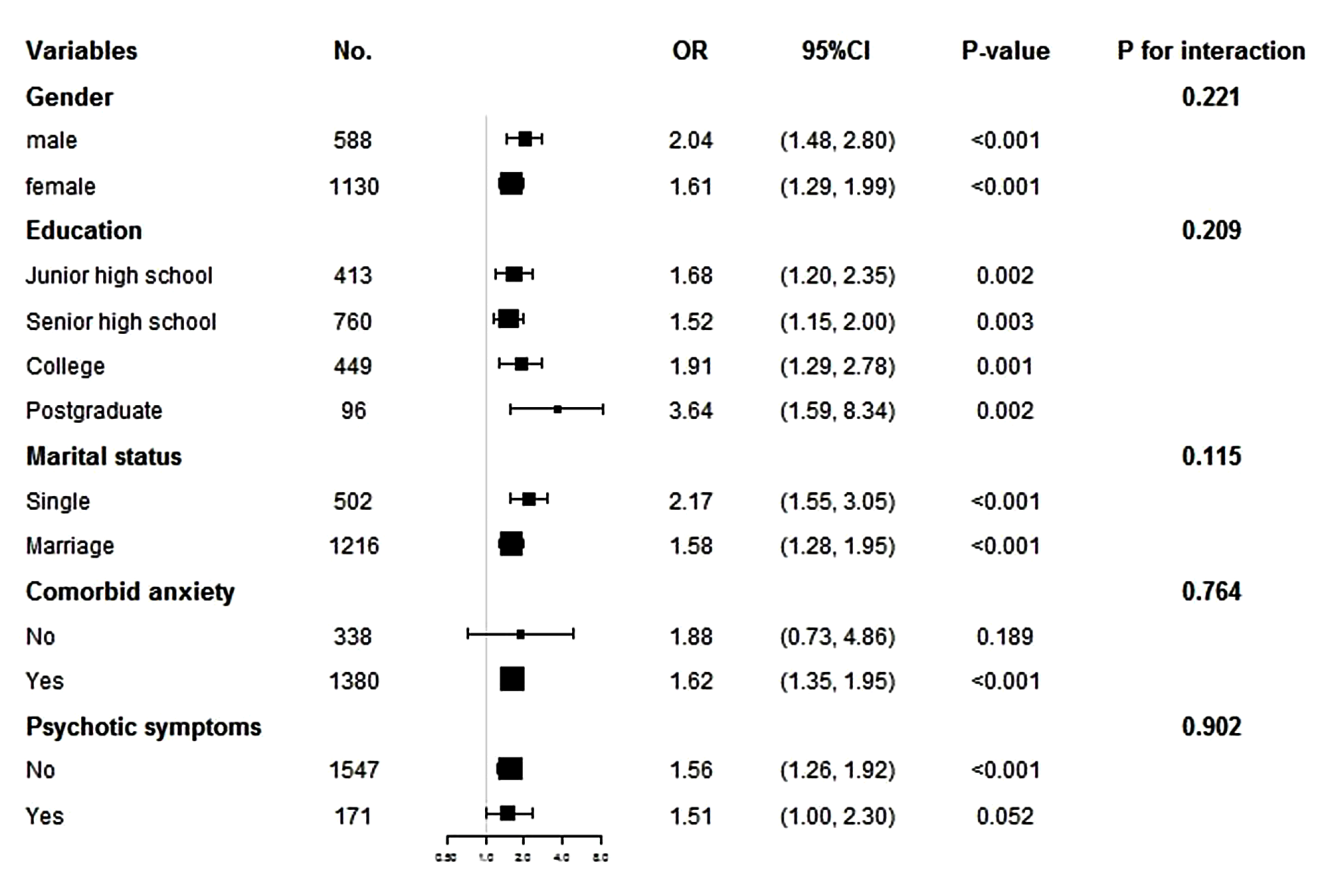
Subgroup analysis of the association between fasting blood glucose and suicide attempts. The OR (95% CI) was derived from the Logistic regression model. (Age, sex, education, duration of illness, HAMD, HAMA, TSH, A-TG, A-TPO, TC, HDL-c, TG, LDL-c, SBP, DBP, and psychotic symptoms were adjusted)

As shown in Fig. 4, subgroup analysis revealed a highly consistent pattern in the following subgroups: gender (male, female), education (junior high school, senior high school, college, postgraduate), marital status (single, married), comorbid anxiety (no, yes), and psychotic symptoms (no, yes) (all P for interaction > 0.05).

## Discussion

To our knowledge, this study is the first to investigate the nonlinear correlation between FBG levels and SA in patients with FEDN MDD in a relatively large sample size. The initial findings of this study demonstrated that FBG levels were positively associated with the presence of suicidal ideation and attempts in Chinese FEDN MDD patients, even after accounting for potential confounding variables. Furthermore, our analysis revealed a U-shaped nonlinear relationship between FBG levels and the risk of SA, with an inflection point occurring at an FBG level of 5.34 mmol/l. Interestingly, we observed that on the left side of the inflection point, lower FBG levels were actually associated with a higher risk of SA, while on the right side of the inflection point, higher FBG levels were associated with a higher risk of SA. These findings provide novel insights into the complex relationship between FBG levels and the risk of SA in patients with MDD.

The findings of this study suggest that there is a consistent link between FBG levels and SA, which has also been emphasized by earlier studies. Emerging research has shown that FBG plays a significant role in estimating the risk of suicide and other suicidal behaviors. Specifically, individuals with MDD may experience abnormal glucose metabolism, including higher levels of glucagon, insulin, and fasting blood sugar [26]. A recently published study by Liu et al. found that MDD patients with suicidal behavior had higher FBG levels compared to MDD patients without suicidal behavior [27]. This correlation suggests that symptoms of depression and anxiety can contribute to an increased risk of suicide by raising blood sugar levels. Another study by Koponen et al. indicated that patients with suicidal thoughts or suicidal attempts had higher blood glucose levels in an oral glucose tolerance test (OGTT), which is used to assess the body’s ability to regulate blood sugar [28]. The correlation between blood sugar levels and suicidal behavior in MDD patients involves various factors, including metabolic, signaling, and genetic aspects. Excessive fluctuations and uncontrolled blood sugar levels can weaken the patient’s self-management awareness, increasing the likelihood of suicidal ideation [29]. On the other hand, suicidal thoughts can also lead to metabolic issues and reduced lipid synthesis, exacerbating insulin resistance and raising blood sugar levels in individuals with type 2 diabetes [30]. Furthermore, MDD patients with suicidal depression tend to have lower serum levels of brain-derived neurotrophic factor (BDNF) compared to the general population. Insufficient levels of BDNF can hinder the body’s glucose metabolism and insulin sensitivity, potentially contributing to the development of insulin resistance [31]. Stress, which is known to disrupt homeostasis, can have significant physiological and behavioral effects. Chronic stress can lead to changes in the function and structure of the cerebral stress system, negatively impacting various brain regulatory functions [26]. The activation of the hypothalamic-pituitary-adrenal (HPA) axis, stimulated by anxiety and depression, results in the release of corticotropin-releasing hormone (CRH) and glucocorticoids. Glucocorticoids, in turn, can cause hyperglycemia by stimulating gluconeogenesis, the process of generating glucose from non-carbohydrate sources. In summary, this expanded explanation highlights the consistent correlation between FBG levels and suicidal behavior, emphasizing the role of symptoms of depression, anxiety, and stress in raising blood sugar levels. It also underscores the importance of considering various metabolic and genetic factors that contribute to the link between blood sugar levels, suicidal ideation, and the development of insulin resistance [33].

Furthermore, the study also determined a U-shaped, nonlinear relationship between FBG and suicidal ideation in patients with MDD. The generalized additive model with a smooth curve revealed that for patients with FBG levels lower than the threshold point of 5.34 mmol/l, there was a negative correlation between FBG level and suicidal ideation. This finding is novel and has not been reported in previous studies. The underlying psychopathological mechanism behind this negative correlation has not been fully established. Previous research has suggested a potential link between depression and hypoglycemia. Zhang et al. found that individuals with type 2 diabetes and depression were more likely to have poor glycemic control and experience hypoglycemia compared to those without depression [34]. This finding held true even when accounting for the comparable use of sulphonylurea and insulin, thus suggesting that depression may be associated with poor self-care and medication nonadherence. Biggers et al. conducted a study in Thailand and demonstrated that depressive symptoms were associated with the frequency of hypoglycemia in individuals with type 2 diabetes [35]. It is important to note that prolonged low blood sugar can cause neuronal damage by depriving neurons of essential oxygen and nutrients, which can have detrimental effects on the nervous system [36]. In such cases, the nervous system may undergo a series of pathological changes [37], including neuronal atrophy, synaptic loss, and myelin damage [38]. Suicide rates in individuals with depression have also been linked to alterations in brain structure and function, particularly in regions involved in emotion regulation and decision-making, such as the frontal-subcortical circuit, the suicide circuit, and the reward circuit [39]. Positron emission tomography (PET) studies of suicide patients have shown differences in the volume and shape of regions such as the prefrontal cortex, cingulate gyrus, hippocampus, and amygdala when compared to non-suicidal control groups [40]. Additionally, reduced metabolic activity in the amygdala and prefrontal cortex has been observed in suicide patients [41, 42]. Research using functional and structural neuroimaging techniques has revealed distinct abnormalities in gray matter volume and neural circuitry in cortical regions, particularly in the prefrontal cortical and dorsal anterior cingulate gyrus, in adults who have attempted suicide compared to both affective and healthy adult controls [43]. In the present study, it was found that both high and low blood glucose levels significantly increased the risk of suicidal ideation in patients with comorbid FEDN MDD. Given the complex relationship between FBG and suicidal ideation, further in-depth research is necessary to elucidate the underlying pathological mechanisms involved.

This study has several limitations. First, the patients in this study were recruited from the Han Chinese psychiatric outpatient department of a general hospital in Taiyuan, Shanxi Province, China. Therefore, it is important to confirm our findings in a population with different ethnic and clinical backgrounds. Second, because this was a cross-sectional study, it could not demonstrate a causal relationship between FBG and SA. Future studies should use a longitudinal study design to more thoroughly explore the causal relationship between FBG and SA in patients with MDD. Third, SA were detected using medical records and interviews, which are unstructured assessment methods for suicidal behavior. In addition, we were not provided with precise information about the plans or thoughts behind the suicide. Consequently, to gather information on these issues related to SA, a specific suicide questionnaire should be used in future studies. Fourth, the MDD patients in this study were having their first episode and had not received any medication for their depressive symptoms. Even though there was an advantage to recruiting first-episode and medication-free patients, we cannot entirely rule out patients whose diagnosis may have changed to bipolar disorder, as the initial depressive episode may have paralleled the MDD episode. Fifth, glycated hemoglobin (HbA1c) provides a more stable and long-term reflection of average blood glucose levels, which could potentially offer additional insights into the glucose management of the subjects. However, we did not measure this data. Future research could benefit from including HbA1c levels to better understand the correlation between chronic glucose control and suicidality in patients with depression. Sixth, the MDD patients encompassed in this study displayed pronounced severity in their depressive symptoms, as evidenced by 17-item HAMD scores of 24 or higher and mean HAMA scores exceeding 20. Consequently, the generalizability of our findings to populations with milder depressive symptoms may be limited. Finally, a number of confounding variables, such as social status, family income, smoking, alcohol consumption, personality traits, and biological factors, may have a significant impact on the association between FBG and SA but were not gathered. To further comprehend the pathophysiological mechanisms behind the association between FBG and SA in MDD patients, future studies should take into account additional confounding variables.

In conclusion, the present investigation delineated a U-shaped correlation between FBG and SA in Chinese patients diagnosed with FEDN MDD, pinpointing an inflection at 5.34 mmol/L. Below this threshold, FBG levels were inversely related to SA, whereas above it, a positive correlation emerged. However, the intricate mechanisms underpinning the relationship between FBG and suicidal behaviors in MDD remain to be fully elucidated, necessitating further inquiry. Moreover, given the inherent limitations of this study—including its cross-sectional nature, the absence of structured diagnostic tools, and incomplete data collection—these findings must be regarded as provisional. Validation through prospective longitudinal studies is imperative to confirm these preliminary observations.

## Data Availability

The datasets used and/or analysed during the current study available from the corresponding author on reasonable request.

## References

[1] Geraets AFJ, Schram MT, Jansen JFA, Koster A, Dagnelie PC, van Greevenbroek MMJ, Stehouwer CDA, Verhey FRJ, Köhler S (2021). The relation of depression with structural brain abnormalities and cognitive functioning: the Maastricht study. Psychol Med.

[2] Li H, Luo X, Ke X, Dai Q, Zheng W, Zhang C, Cassidy RM, Soares JC, Zhang X, Ning Y (2017). Major depressive disorder and suicide risk among adult outpatients at several general hospitals in a Chinese Han population. PLoS ONE.

[3] Harwood D, Hawton K, Hope T, Jacoby R (2001). Psychiatric disorder and personality factors associated with suicide in older people: a descriptive and case-control study. I J Geriatr Psychiatry.

[4] Kim SW, Stewart R, Kim JM, Shin IS, Yoon JS, Jung SW, Lee MS, Yim HW, Jun TY (2011). Relationship between a history of a suicide attempt and treatment outcomes in patients with depression. J Clin Psychopharmacol.

[5] Zhao K, Zhou S, Shi X, Chen J, Zhang Y, Fan K, Zhang X, Wang W, Tang W (2020). Potential metabolic monitoring indicators of suicide attempts in first episode and drug naive young patients with major depressive disorder: a cross-sectional study. BMC Psychiatry.

[6] Nock MK, Hwang I, Sampson NA, Kessler RC (2010). Mental disorders, comorbidity and suicidal behavior: results from the National Comorbidity Survey Replication. Mol Psychiatry.

[7] Zhou Y, Ren W, Sun Q, Yu KM, Lang X, Li Z, Zhang XY (2021). The association of clinical correlates, metabolic parameters, and thyroid hormones with suicide attempts in first-episode and drug- naïve patients with major depressive disorder comorbid with anxiety: a large-scale cross-sectional study. Transl Psychiatry.

[8] May AM, Czyz EK, West BT (2020). Differentiating adolescent suicide attempters and ideators: a classification tree analysis of risk behaviors. J Adolesc Health.

[9] Hauser M, Galling B, Correll CU (2013). Suicidal ideation and suicide attempts in children and adolescents with bipolar disorder: a systematic review of prevalence and incidence rates, correlates, and targeted interventions. Bipolar Disord.

[10] Perez J, Beale E, Overholser J, Athey A, Stockmeier C (2022). Depression and alcohol use disorders as precursors to death by suicide. Death Stud.

[11] Rhee TG, Shim SR, Forester BP, Nierenberg AA, McIntyre RS, Papakostas GI, Krystal JH, Sanacora G, Wilkinson ST (2022). Efficacy and safety of ketamine vs electroconvulsive therapy among patients with major depressive episode: a systematic review and meta-analysis. JAMA Psychiatry.

[12] Ceretta LB, Réus GZ, Abelaira HM, Jornada LK, Schwalm MT, Hoepers NJ, Tomazzi CD, Gulbis KG, Ceretta RA, Quevedo J (2012). Increased prevalence of mood disorders and suicidal ideation in type 2 diabetic patients. Acta Diabetol.

[13] Batty GD, Kivimaki M, Park IS, Jee SH (2012). Diabetes and raised blood glucose as risk factors for future suicide: cohort study of 1 234 927 Korean men and women. J Epidemiol Community Health.

[14] Kim YC, Um YH, Kim SM, Kim TW, Seo HJ, Hong SC, Jeong JH (2022). Suicide risk in patients with diabetes varies by the duration of diabetes: the Korea National Health and Nutrition Examination Survey (2019). Psychiatry Investig.

[15] Ye G, Li Z, Yue Y, Wu Y, Yang R, Wang H, Wu S, Zhou Y, Zhao X, Lv X, Yuan N, Li R, Zhang G, Ganapathi PB, Wu HE, Du X, Zhang XY (2022). Suicide attempt rate and the risk factors in young, first-episode and drug-naïve Chinese Han patients with major depressive disorder. BMC Psychiatry.

[16] Sublette ME, Milak MS, Galfalvy HC, Oquendo MA, Malone KM, Mann JJ (2013). Regional brain glucose uptake distinguishes suicide attempters from non-attempters in major depression. Arch Suicide Res.

[17] Li XY, Tabarak S, Su XR, Qin Z, Chai Y, Zhang S, Wang KQ, Guan HY, Lu SL, Chen YN, Chen HM, Zhao L, Lu YX, Li SX, Zhang XY (2021). Identifying clinical risk factors correlate with suicide attempts in patients with first episode major depressive disorder. J Affect Disord.

[18] Ma YJ, Wang DF, Yuan M, Zhang XJ, Long J, Chen SB, Wu QX, Wang XY, Patel M, Verrico CD, Liu TQ, Zhang XY (2019). The prevalence, metabolic disturbances and clinical correlates of recent suicide attempts in Chinese inpatients with major depressive disorder. BMC Psychiatry.

[19] Dong R, Haque A, Wu HE, Placide J, Yu L, Zhang X (2021). Sex differences in the association between suicide attempts and glucose disturbances in first-episode and drug naïve patients with major depressive disorder. J Affect Disord.

[20] Shangguan F, Chen Z, Feng L, Lu J, Zhang XY (2022). The prevalence and clinical correlates of suicide attempts in comorbid subclinical hypothyroidism in patients with never-treated major depressive disorder in China. J Affect Disord.

[21] Platt S, Bille-Brahe U, Kerkhof A, Schmidtke A, Bjerke T, Crepet P, De Leo D, Haring C, Lonnqvist J, Michel K (1992). Parasuicide in Europe: the WHO/EURO multicentre study on parasuicide. I. introduction and preliminary analysis for 1989. Acta Psychiatr Scand.

[22] Lin J, Wang X, Dong F, Du Y, Shen J, Ding S, Wang L, Ye M, Wang Y, Xia N, Zheng R, Chen H, Xu H (2018). Validation of the Chinese version of the Hamilton Rating Scale for Depression in adults with epilepsy. Epilepsy Behav.

[23] Yang W, Zhang G, Jia Q, Qian ZK, Yin G, Zhu X, Alnatour OI, Trinh TH, Wu HE, Lang X, Du X, Zhang X (2019). Prevalence and clinical profiles of comorbid anxiety in first episode and drug naïve patients with major depressive disorder. J Affect Disord.

[24] Shen Y, Wei Y, Yang XN, Zhang G, Du X, Jia Q, Zhu X, Ma Y, Lang X, Luo X, Zhang XY (2020). Psychotic symptoms in first-episode and drug naïve patients with major depressive disorder: prevalence and related clinical factors. Depress Anxiety.

[25] Kernan WN, Viscoli CM, Brass LM, Broderick JP, Brott T, Feldmann E, Morgenstern LB, Wilterdink JL, Horwitz RI (2000). Phenylpropanolamine and the risk of hemorrhagic stroke. N Engl J Med.

[26] Detka J, Kurek A, Basta-Kaim A, Kubera M, Lasoń W, Budziszewska B (2014). Elevated brain glucose and glycogen concentrations in an animal model of depression. Neuroendocrinology.

[27] Liu W, Wu Z, Sun M, Zhang S, Yuan J, Zhu D, Yan G, Hou K (2022). Association between fasting blood glucose and thyroid stimulating hormones and suicidal tendency and disease severity in patients with major depressive disorder. Bosn J Basic Med Sci.

[28] Koponen H, Kautiainen H, Leppanen E, Mantyselka P, Vanhala M (2015). Association between suicidal behaviour and impaired glucose metabolism in depressive disorders. BMC Psychiatry.

[29] Li C, Lumey LH (2019). Impact of disease screening on awareness and management of hypertension and diabetes between 2011 and 2015: results from the China health and retirement longitudinal study. BMC Public Health.

[30] Russell KS, Stevens JR, Stern TA (2009). Insulin overdose among patients with diabetes: a readily available means of suicide. Prim Care Companion J Clin Psychiatry.

[31] Marano CM, Workman CI, Lyman CH, Kramer E, Hermann CR, Ma Y, Dhawan V, Chaly T, Eidelberg D, Smith GS (2014). The relationship between fasting serum glucose and cerebral glucose metabolism in late-life depression and normal aging. Psychiatry Res.

[32] Kalantaridou SN, Zoumakis E, Makrigiannakis A, Lavasidis LG, Vrekoussis T, Chrousos GP (2010). Corticotropin-releasing hormone, stress and human reproduction: an update. J Reprod Immunol.

[33] Fagerholm V, Haaparanta M, Scheinin M (2011). α2-adrenoceptor regulation of blood glucose homeostasis. Basic Clin Pharmacol Toxicol.

[34] Zhang Y, Ting RZ, Yang W, Jia W, Li W, Ji L, Guo X, Kong AP, Wing YK, Luk AO, Sartorius N, Morisky DE, Oldenburg B, Weng J, Chan JC (2015). China Depression in Chinese patients with type 2 diabetes (DD2) Study Group. Depression in Chinese patients with type 2 diabetes: associations with hyperglycemia, hypoglycemia, and poor treatment adherence. J Diabetes.

[35] Biggers A, Sharp LK, Nimitphong H, Saetung S, Siwasaranond N, Manodpitipong A, Crowley SJ, Hood MM, Gerber BS, Reutrakul S (2019). Relationship between depression, sleep quality, and hypoglycemia among persons with type 2 diabetes. J Clin Transl Endocrinol.

[36] Camandola S, Mattson MP (2017). Brain metabolism in health, aging, and neurodegeneration. EMBO J.

[37] López-Gambero AJ, Martínez F, Salazar K, Cifuentes M, Nualart F (2019). Brain glucose-sensing mechanism and energy homeostasis. Mol Neurobiol.

[38] Mohseni S (2001). Hypoglycemic neuropathy. Acta Neuropathol.

[39] Phillips ML, Drevets WC, Rauch SL, Lane R (2003). Neurobiology of emotion perception I: the neural basis of normal emotion perception. Biol Psychiatry.

[40] Miller JM, Hesselgrave N, Ogden RT, Sullivan GM, Oquendo MA, Mann JJ, Parsey RV (2013). Positron emission tomography quantification of serotonin transporter in suicide attempters with major depressive disorder. Biol Psychiatry.

[41] New AS, Goodman M, Triebwasser J, Siever LJ. Recent advances in the biological study of personality disorders. Psychiatr Clin North Am. 2008;31(3):441 – 61, vii. 10.1016/j.psc.2008.03.011. 10.1016/j.psc.2008.03.011 18638645

[42] Auerbach RP, Pagliaccio D, Allison GO, Alqueza KL, Alonso MF (2021). Neural correlates associated with suicide and nonsuicidal self-injury in youth. Biol Psychiatry.

[43] Martin PC, Zimmer TJ, Pan LA (2015). Magnetic resonance imaging markers of suicide attempt and suicide risk in adolescents. CNS Spectr.

